# Ten priorities for national brain and mental health plans

**DOI:** 10.3325/cmj.2019.60.152

**Published:** 2019-04

**Authors:** François Mauguière, Jose-Luis Trejo, Pavle Andjus, Cristina Vergara, Roland Pochet

**Affiliations:** 1 French Brain Council, Paris, France; 2 Spanish Brain Council, Madrid, Spain; 3 Serbian Brain Council, Belgrade, Serbia; 4 Université Libre de Bruxelles, Brussels, Belgium; 5 Belgian Brain Council, Brussels, Belgium *rpochet@ulb.ac.be*

National Brain Councils (NBCs) are independent, multidisciplinary units gathering patients’ associations, scientific societies in the fields of neuroscience, psychiatry, neurology, and neurosurgery, as well as pharmaceutical and medical device industry, in each country. Their aim is to speak in one strong voice on behalf of the whole “brain space.” Although NBCs are independent, they have aligned main goals, which include improving the life quality of people with a neurological or mental disorder, raising awareness of these disorders, stimulating brain related research, fostering the exchange between different disciplines and associations, and lobbying institutions and governments for enhanced research and treatments.

While the organization, management, and funding of health services fall within the exclusive competence of national governments, the European Union (EU) is required to protect human health across all policy areas and to work with EU countries to improve public health, prevent human illness, and eliminate the sources of danger to physical and mental health. Moreover, the Consolidated version of the Treaty on European Union and the Treaty on the Functioning of European Union (1) encourage greater involvement of national parliaments.

A core group of NBCs proposed that all NBCs contribute to the involvement of national parliaments. To address such a challenge, the 4th NBCs Academy held in Lisbon, April 2018 (2), defined NBCs' action and decided to conduct the “Survey on the Current State of Care for Patients Suffering from Brain Diseases.” The questionnaire was created in Google Forms and consisted of 32 questions (10 main questions and 22 sub-questions) with three possible answers (yes/no/no answer). It was answered by NBCs representatives from 17 countries (Belgium, Bulgaria, Croatia, France, Germany, Greece, Ireland, Latvia, Luxemburg, Malta, Netherlands, Norway, Poland, Portugal, Serbia, Spain, Turkey) (Table 1). Responses clearly show that despite differences between health care organizations, representatives from all NBCs recognized the same issues related to brain disorders management and reached a consensus on 10 main priorities for brain health.

**Table 1:** Survey on the current state of care for patients suffering from brain diseases – questions and selected responses by representatives of National Brain Councils

Survey question	Selected responses
1. Do you agree with the current state of prevention being A. not homogeneous within regions B. of good quality of care except for psychiatry	*Netherlands*: In 2015, the Dutch Healthcare system was organized in such a way that municipalities were made responsible for the overall health care budget in their city/region. This has led to many problems: counselors not sufficiently prepared for the task, not knowledgeable of disease specifics, more bureaucracy for family doctors, mistakes, etc. This goes for the whole field of brain disorders. The quality of care differs per city region. *Poland*: There is no prevention at all, either psychiatric or neurological, except for stroke, which is recognized mostly as a cardiovascular disease.
2. Do you agree with the current state of diagnosis? A. In general delay in accurate diagnosis B. Delay in diagnosis for neurocognitive and sleep disorders	*Belgium:* The early detection should be integrated in the educational program for students... Besides, often not enough opportunities are given for the access to diagnostic devices…. *France*: The impact of sleep disorders on global health and on risk of neurodegenerative diseases should be emphasized. Diagnostic rates should be improved at early stages of neurocognitive disorders.
3. Do you agree with the current state of treatment A. Deficiency in multidisciplinary integrated home care B. Weaknesses in rehabilitation program and nonpharmacological therapeutics	*Norway*: Specialized rehabilitation has been underprioritized in the last few years, with responsibilities being shifted toward the community level. Cognitive rehabilitation programs are sparse/lacking. *Belgium*: Unfortunately, because of political goals, the main targets are often to reduce the health cost in the short term, and contrary to promises made, not enough money is given for these two points.
4. Do you agree with the order of priorities presently allocated?	*France*: Epilepsy, headache, and sleep disorders should be included on the list of priorities for which allocated resources are presently deficient. Psychiatric diseases as a whole is a too generic term to qualify specific diseases (depression, schizophrenia, obsessive-compulsive disorders, autism…) affecting 38% of the population, representing the first cause of disability-adjusted life years (DALYs) (20% in comparison to 5% for neurological disorder), which are the first cause of costs and will in 2020 be the first cause of world handicap. *Belgium*: Migraine, the most prevalent neurological disorder and the second most disabling in DALYs after stroke, is in a great need of adequate support.
5. Do you agree with the existence of the following current trends for improvement of patients care? A. National policy to reduce hospitalisation by improving health care pathways and ambulatory management B. National center for better diagnosis of rare neurodiseases	*Belgium*: National centers are certainly a good idea for the best efficacy, but we have to separate the diagnostic centers and care centers; we have to preserve the accessibility of the efficient care for all the people in the whole country, not just in towns or near the universities.
6. Do you agree with the following increases in your government spending for patients with brain disease: A. Initiatives with budget for Huntington diseases in particular home care B. Initiatives with budget for multiple sclerosis in particular in home care C. Initiatives for amyotrophic lateral sclerosis in particular in home care D. Existence of the National Plan for Alzheimer and Related disorders (ADRD in France) E. Existence of plans for rare diseases F. Initiatives with budget for autism	*Portugal:* Those initiatives exist, are significant but not sufficient. *Belgium*: Yes, but all the patients suffering from a brain disease need those initiatives.
7. About the efforts made to increase collaboration among medical (general practitioners, neurologists, and psychiatrists) and paramedical staff (GPs, nurses, other trained assistants) for the care of patients: A. Do they exist? B. Are they efficient? C. Exist only among patients associations	*Belgium*: Good cooperation between patients' organizations and hospitals/care homes that have signed the conventions but not enough resources to act efficiently. *Norway*: The government initiated a reform a few years ago, called the Collaboration Plan, to improve the collaboration between the first and secondary health care. However, as there are no budget allocations or extra time for such efforts, the actual collaboration on an individualized level as well as the more general work, is quite fragmented and depends much upon the individual specialist and GP.
8. Access to new and innovative technologies (neuroimaging, PET scan, clinical neurophysiology platforms): A. Have this access improved in general? B. For multiple sclerosis? C. For stroke? D. Shortening approval process for drugs to be on the market? E. Reimbursement for accessing new technologies? F. Access to new disease modifying drugs? G. Access to non pharmacological approaches?	*France*: Approval of reimbursement for innovative technologies is a very slow process (for instance magnetoencephalography in epilepsy, the use of innovative tracers for PET in psychiatric diseases, Parkinson disease, and epilepsy is supported by clinical research programs for years before being considered for reimbursement by national health insurance system). *Netherlands*: Generally speaking, this is an area of discussion and conflict. The health insurance companies have a say in this as well, which often conflicts with the hospital views. Stroke care is rather well organized in bigger cities but not in smaller hospitals. New medicines often are too expensive and only given to highly selected patients.
9. Is the implementation for better coordination in the care of brain diseases still left behind?	*Netherland*s: Yes, the whole field of brain disorders is still lagging behind in comparison to eg, diabetes, cancer, etc.
10. Do you agree with the following challenges to be performed/tackled for improving care for patients with brain diseases: A. Develop evidence-based and socially responsible policy by collecting data from ongoing clinical trials and basic research to optimally address unmet needs B. Develop translational research C. Increase education on innovative technologies D. Improve structure of coordinated health care E. Improve rehabilitation programmes F. Improve budget and research in psychiatry	*France*: The impact of psychiatric diseases on global health in France has been underevaluated. Therefore, the French Brain Council proposes that the term “Brain and Mental Health Plan” be preferred to that of “Brain Plan” to build up the forthcoming plan at the national and European levels. *Portugal*: National and EU initiatives should draw attention to these challenges.

## THE 10 PRIORITIES

1. To increase the budget devoted to basic and clinical brain research, in particular in psychiatric diseases

2. To improve detection, prevention, and treatment of brain disorders, in particular psychiatric diseases; to facilitate the access to dedicated acute care of stroke; and to increase the homogeneity of prevention and early treatment between regions

3. To shorten the diagnostic delay for brain disorders; in particular through the development of biomarkers crucial for clinical testing of new treatments at early stages of neurodegenerative diseases

4. To develop multidisciplinary home care and rehabilitation programs and provide funding for the authorities responsible for the delivery of these programs

5. To facilitate collaboration among general practitioners, neurologists, psychiatrists, psychologists, geriatricians, nurses, and paramedical staff

6. To involve patients' organizations in the coordination of care for brain disorders

7. To develop and disseminate evidence-based recommendations and socially responsible policy, based on ongoing clinical trials and basic research

8. To foster translational research programs with clear outcomes for patients and evaluate them through their final impact on patients’ quality of life and on healthy brain aging of the general population

9. To facilitate patients’ access to innovative technologies and new disease-modifying drugs by shortening the approval delay for reimbursement by national health insurance systems

10. To increase the education on innovative technologies, including digital medicine, bioengineering, and genome screening

These 10 priorities, validated by a consensus after a second reading by all NBC representatives, show some similarities to the recommendations expressed in the 2018 Organisation for Economic Co-operation and Development-EU report “Health at a Glance: Europe” (3), thus validating the bottom-up approach that was used in their identification. NBCs will take the opportunity of the 5th Academy of NBCs in Dubrovnik in May 2019, before Croatia assumes the EU Presidency, to disseminate these priorities among the EU national parliaments to help them define their health policies.
